# Prevalence, pattern and factors associated with ocular disorders in small-scale welders in Katwe, Kampala

**DOI:** 10.1186/s12886-019-1150-x

**Published:** 2019-07-10

**Authors:** Immaculate Atukunda, Rebecca Claire Lusobya, Samia Hersi Ali, John Mukisa, Juliet Otiti-Sengeri, C. Ateenyi-Agaba

**Affiliations:** 1 Department of Ophthalmology, Makerere University College of Health Sciences, and Mulago National Referral Hospital, P.0.BOX, 7072 Kampala, Uganda; 2 Clinical Epidemiology Unit, School of Medicine, Makerere University College of Health Sciences, Kampala, Uganda

**Keywords:** Welders, Ocular disorders, Patterns, Associated factors

## Abstract

**Background:**

Welders are at an increased risk of eye disorders as a result of their occupation, leading to enormous vocational and economic consequences. With limited published studies among welders in low resource settings, we sought to determine the prevalence, pattern and factors associated with ocular disorders among small-scale welders in Katwe, Kampala.

**Methods:**

In a field-based cross-sectional study, we recruited 343 small-scale welders. Simple random sampling was done to select the study participants. A pretested questionnaire was used to collect information on demographics, ocular, general history, systemic and ocular examination. The proportion of small-scale welders with ocular disorders (defined as any abnormal finding on eye examination) was determined. The bivariate and multivariate analyses were carried out, using logistic regression methods at a level of significance of 0.05.

**Results:**

The mean age of the participants was 36 years (SD ± 12). The overall prevalence of ocular disorders was found to be 59.9%. The common ocular disorders included conjunctiva disorders (32%) and presbyopia (27%). There was a statistically significant relationship between females (OR = 4.279, *P*-value = 0.007), age 35 and above (OR = 4.244, *P*-value< 0.001), history of foreign body removal (OR = 1.677, *P*-value = 0.041), and ocular disorders.

**Conclusions:**

There is a high prevalence of ocular disorders among small-scale welders. Conjunctiva disorders, presbyopia and myopia were the commonest. Being female, age 35 and above and foreign body removal, were significantly associated with ocular disorders among welders. Policies should be put in place to ensure all welders use proper personal -protective equipment (welding helmets), and also receive regular eye checkup and health education.

**Electronic supplementary material:**

The online version of this article (10.1186/s12886-019-1150-x) contains supplementary material, which is available to authorized users.

## Background

Welding is a growing industrial process, widely used for the permanent joining of metal parts. The more common welding processes can be classified as arc welding, gas, resistance, energy beam and solid-state welding [1]. Welders are constantly exposed to the risk of ocular injuries and disorders from their profession [2], because welding is one of the highest artificial sources of visible and invisible optical radiation [3, 4]. The adverse effects of welding stem from the prolonged exposure to visible light rays, infrared and ultraviolet (UV) radiation, all of which are emitted in various degrees, by various types of welding [5, 6]. The long-term exposure to ultraviolet radiation is associated with conditions such as pterygia, pingueculae, keratopathy, maculopathy [7], and eye irritation, among others [8]. Other consequences of emissions from welding include: photo keratoconjunctivitis, chronic actinic keratopathy, photophthalmia, and corneal opacity, which may be permanent and sight-threatening [1, 3]. Welders are also at a high risk of eye injuries such as traumatic mydriasis, traumatic iritis and foreign bodies, due to flying metal chips and thermal burns [9, 10]. The prevalence of these disorders is higher in developing countries, due to the poor organization and policy implementation regarding occupational safety [11–13]. For example, with the increased industrialization in Kampala city, Uganda has witnessed an increase in small-scale industries; thus, an increase in welding practice and occupational eye diseases [14]. The latter may be very disabling, and most frequently in the active and most productive years of life; consequently leading to enormous vocational and economic consequences to the welders, and the health system [15].

The mainstay of ocular protection from welding arc radiation is an infrared absorbing green glass filter, placed within the welder’s helmet [16]. The helmet also offers further protection from mechanical injury [17]. Unfortunately, workers don’t always wear goggles or safety glasses, because of low perception of risk, poorly maintained lenses, discomfort, having to wear prescription lenses underneath, and vanity [13, 18]. The lack of knowledge on the use of protective gear during welding further fuels the welders’ non-compliance [17]. Uganda, like many other developing countries, does not have a law or policy that enforces the use of protective wear, especially among the small-scale welders.

A previous study done in Nigeria to determine the pattern of non-traumatic ocular disorders, found a prevalence of Ocular disorders to be 66.4% of the eyes [19]. To our knowledge, no similar published studies have been documented in East Africa. Studies in other settings have highlighted that a longer duration of welding, eye irritation by fumes from welding, lack of Personal Protective Equipment (PPE), or insufficient use of this equipment, carelessness or accidents at work, fatigue caused by overworking, lack of modern equipment and lack of skill or inexperience have been associated with increased risk of ocular disorders among welders [7, 11, 14, 20].

However, there is limited published literature on the burden, pattern and associated factors of ocular disorders in Uganda. The findings from the study would provide evidence for the development and strengthening of eye health policies among small scale welders in Uganda and the other low income countries.

## Methods

### Study setting and population

This study was a field-based descriptive cross-sectional study, of small-scale welders in Katwe, Kampala, Uganda, carried out between 1st April and 31st August 2016. All the potential study participants were approached at their work stations; informed consent obtained and underwent study evaluations. Katwe is located in Makindye division, approximately 3 km by road, from Kampala’s central business district. It consists of two big parishes namely; Katwe 1 (10 zones) and Katwe 2 (9 zones). Katwe 1 is a commercial place, where all kinds of businesses and activities take place (most welding workshops are located here), while Katwe 2 is a slum-residential place, where the workers reside. A preliminary survey showed that Katwe has the highest number of small-scale welders in Kampala. It has over 3000 artisans and metal fabricators, in over 800 individual small enterprises, with welders accounting for up to half of these. The workshops of the small-scale welders are mainly located by the roadside.

The study enrolled adult small scale welders working in Katwe, Kampala, during the study period, who had given consent. Welders younger than 18 years, who were found working at the welding stations, were also included and considered as emancipated minors. This was per the permission granted by the Ethical committee. We excluded small scale welders with known allergies to drops or stains used in the study for the ocular examination.

### Sample size

The sample size for prevalence and pattern of ocular disorders was determined using the Kish and Leslie’s formula [21]. We considered a standard normal Z score corresponding to the 95% confidence interval (1.96), a precision of 5% and an estimated prevalence of 66.4%, based on the study done in Nigeria. This was to determine the prevalence and pattern of ocular disorders among small-scale welders [19]. A total sample size of 343 participants was obtained. To estimate the sample size for the associated factors, two proportions were used. N = (Z_1_ + Z_2_)^2^2P (1-P)/(P_2_-P_1_)^2^; where: N = Sample size required, Z_1_ is Z value at 95% level of significance = 1.96, Z_2_ is Z value at 80% power = 0.84, and P_1_ is proportion of welders using protective eyewear, who sustain ocular disorders. P_2_ is the proportion of welders not using protective eyewear, who sustain ocular disorders. P = (P_1_+ P_2_)/2. From a study done in Nigeria: P_1_ = 15.5% and P_2_ = 62.5% [11] P = (0.155 + 0.625)/2 = 0.39, N = (1.96 + 0.84)^2^ × 2 × 0.39(1–0.39)/(0.625–0.115)^2^ = 16.9. Therefore, the estimated sample size was 17 people. An adequate sample size of 343 people would better answer all objectives of this study; therefore it is what was used.

### Sampling of the study population

Simple random sampling was done to identify possible study participants. Katwe has approximately 400 welding stations, with each welding station having a minimum of 3 welders. To obtain a sample size of 343, the number of stations considered were 343÷3 = 115 stations. With the help of local council leaders, all the welding stations were labelled with consecutive numbers from 1 to 400.Numbers were written on cards and put in a box. About 115 cards (with the number of welders listed) were picked at random without replacement and these were the ones included in the study. If a sampled welder declined to be interviewed or examined, another number was picked at random. We started study assessments at the first chosen work station in the study area.

### Data collection and ocular assessments

A pretested structured questionnaire (Additional file 1) [22], administered by the researcher or trained research assistants (Ophthalmology clinical officers and nurses), was used for data collection. Participants’ demographic characteristics, relevant welding, social and ocular history were collected. A detailed ocular examination was done at makeshift on-site ocular research clinics in the study area, starting with the right eye then left eye. This included: distance visual acuity using a 6 m Snellen’s chart or illiterate E chart; those with visual acuity (V/A) less than 6/6 were reassessed with a pinhole and then refracted with an autorefractometer. Near vision was then assessed using a Jaeger chart, and then refraction was done on all participants with impaired near vision. Visual fields were assessed by the confrontational method compared with the examiner (the examiner had normal visual fields confirmed by perimetry). Extra ocular muscle activity was assessed; the cover-uncover test was done to assess for phorias. Diplopia was sought for in all directions of the gaze. Amsler grid was done in all subjects, to assess macular function. Examination of the lids, conjunctiva, cornea, anterior chamber, pupil and iris was done using portable slit lamp. Tonometry using Perkin’s applanation tonometer, after instilling anaesthetic drop (tetracaine) and staining the tear film with fluorescein strips, was carried out on all respondents. Dilating of the pupil was done using cyclopentolate eye drops and then indirect ophthalmoscopy performed in study participants with a visual acuity less than 6/6. Any ocular anomaly detected during the patient assessment was both documented and managed where possible, or the relevant specialist was consulted on the course of management and referral.

All completed questionnaires were entered into Epidata version 3.1, with programmed quality control checks.

### Statistical analysis

Descriptive statistical measures such as means, standard deviations and medians, interquartile range, frequencies, proportions, and percentages for continuous and categorical variables wherever appropriate, were computed. The prevalence of ocular disorders (defined as any abnormality found on eye examination) among small-scale welders was determined, with the numerator as the number of welders with ocular disorders, and denominator as the total number of welders enrolled in the study.

At bivariate analysis, logistic regression methods were used, with the outcome dichotomized as *yes = 1,* if one had an ocular disorder, and *no = 0,* if one had no ocular disorder. Variables with a *P*-value of ≤0.2 at bivariate analysis and clinical significance were considered for the stepwise multivariate model. The odds ratios and their 95% confidence intervals were calculated. All results were considered significant, if the P-value was at 0.05 or less. Statistical analysis was performed using STATA 13.0, College Station, Texas, USA.

### Quality control

In order to minimize errors in the process of data collection, patient examination was done by the principal researcher (experienced in ocular examinations). Research assistants were trained on how to take demographics, welding, social and ocular history data, and proper recording of study variables. A daily check on data collection tools was done, to ensure their completeness, accuracy and consistency.

## Results

### Baseline characteristics of the study population

A total of 344 small-scale welders participated in the study, with age ranging from 13 to 78 years, and a mean age of 36 years (SD ± 12). Majority of the welders (95%), were using Electric Arc as the type of welding. About 38% of the welders had spent not more than 5 years in the business, while 35% were more than 10 years in. About 89% of the participants were using protective eye wears (Table 1).

**Table 1:** Table showing the demographic, welding and eye protective wearing characteristics of participants

Variables	Frequency(*N* = 344)	Percentage (%)
Age		
13–25	72	21
26–35	108	32
36–45	84	24
> 45	80	23
Mean(±SD) age of welders	36 (±11.90) years	
Gender		
Male	310	90
Female	34	10
Educational level		
None	30	9
Primary	133	39
Secondary	157	46
Tertiary	24	7
Duration of Welding		
6–10	91	27
> 10	123	35
Mean(±SD) duration of welding	10.87 (±8.90) years	
History of ocular trauma		
Yes	228	66
No	116	34
Agent causing injury		
Flying metal chips	179	79
Flames	47	2
Others	2	1
Injury happen while using protective eye wear		
Yes	32	14
No	196	86
Type of protective eye wear used		
Filters	7	2
Glasses	317	97
Others	4	1
Spectacle correction		
Yes	12	3
No	332	97
Do you use your Spectacle correction? During welding		
Yes	7	2
No	337	98
Foreign body removal		
Yes	186	54
No	158	46

### Prevalence of ocular disorders

**Fig. 1:**
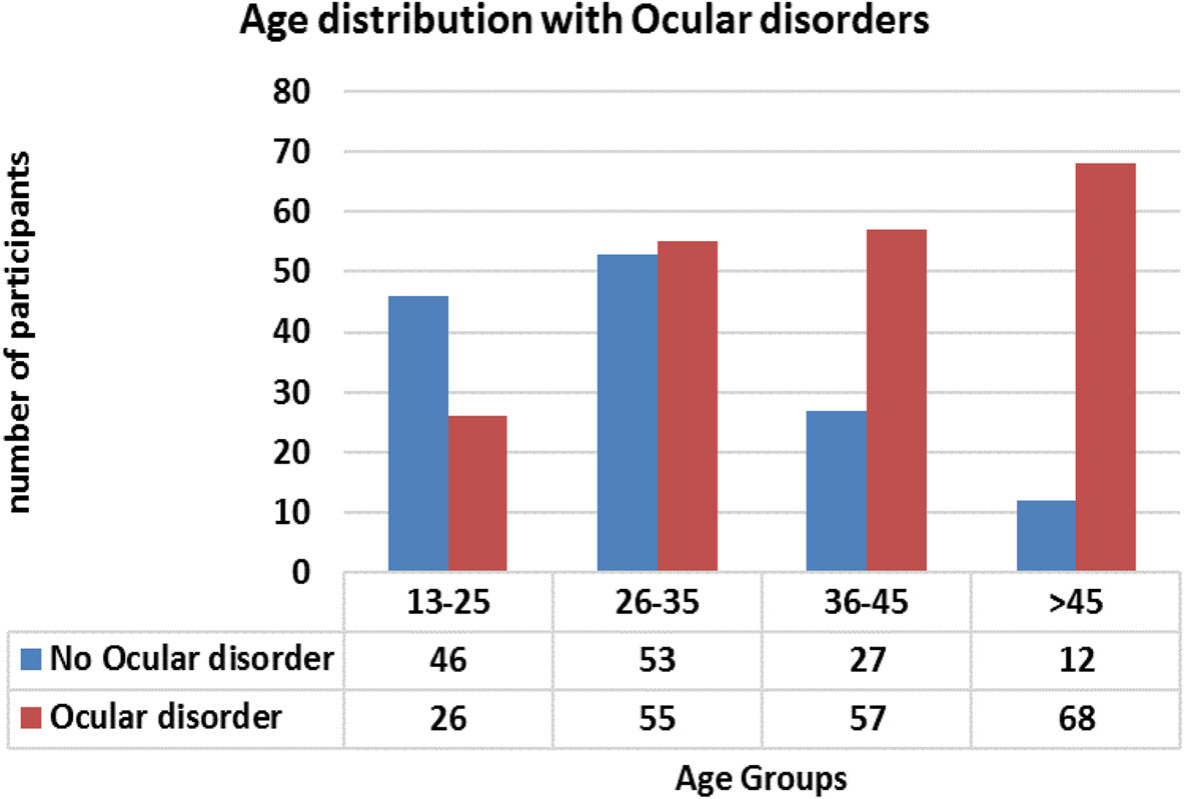
Age distribution of study participants with ocular disorders

In this study, the overall prevalence of ocular disorders among the small-scale welders was 59.9% (95%CI, 54.59–64.96) (Table 2).> The proportion of welders with ocular disorders increased with age; from 36.1% in the 13–25 age range, to 85.0% in the 45 years and above age range (Fig. 1).

**Table 2:** Table showing the prevalence of ocular disorders among welders

	Frequency	Prevalence	[95% Conf. Interval]
No ocular disorder	138	40.12	35.04–45.41
Ocular disorder	206	59.88	54.59–64.96

### Pattern of ocular disorders

About 5.3% (11/206) of the study participants had raised intraocular pressure, and 10.2% (21/206) had myopia With Pingueculum, it was 22.3% (46/206) and 18.0% (37/206) for injection of the eye, who were the commonest eye disorders among the study participants. The rest of the patterns are as shown (Table 3).

**Table 3:** Pattern of ocular disorders in the participants

Disorder	Number of individuals
Visual acuity	
Moderate visual impairment	3
Severe visual impairment	2
Blindness	2
Abnormal stereopsis	3
Abnormal Right visual field	5
Abnormal Extra ocular muscle activity	4
Diplopia	1
Abnormal Intraocular pressure (> 21 mmHg)	11
Refractive error	
Myopia	17
Hypermetropia	1
Presbyopia	94
Refractive Error	
Myopia	21
Hypermetropia	1
Whole Globe	
Phthisis	1
Removed	1
Eye Lids	
Cyst	1
Cyst Upper Lid	1
Moluscum Contangiosum	1
Conjunctiva	
Discharge	3
Dry Eye	5
Injection	37
Papillae	16
Pingueculum	46
Cornea	
Keratitis	3
Tear with stitch	1
corneal scar	4
Erosion	3
Opacity	5
Trantas spots	1
Lens	
Total opacity	11
Pseudophakia	9
Retina	
Hypertensive Retinopathy	2
Pale	1

### Factors associated with ocular disorders among small-scale disorders

At bivariate analysis (Table 4), the factors that were independently associated with increased odds of having an ocular disorder included sex of the welder and age.

The female welders were 4.4 times more likely to have an ocular disorder, as compared to the male counterparts (OR = 4.36, *P*-value = 0.003). Welders, 36 years and above, were more likely to have an ocular disorder.

**Table 4:** Factors associated with ocular disorders among small scale welders

Variable	No disorder *n* (%)	Ocular disorder *n* (%)	OR (95% CI)	*p*-value
Sex				
Male	133(43)	177(57)	1	
Female	5(15)	29(85)	4.36(1.64–11.55)	0.003*
Age				
13–25	46(64)	26(36)	1	
26–35	53(49)	55(51)	1.84(0.99–3.38)	0.051
36–45	27(32)	57(68)	3.74(1.92–7.26)	< 0.001*
≥ 45	12(15)	68(85)	10.03(4.59–21.86)	< 0.001*
Education level				
none/primary	58(36)	105(64)	1	
secondary/tertiary	80(44)	101(56)	0.70(0.45–1.08)	0.104
Type of welding				
Gas flame	7(41)	10(59)	1	
Electric Arc	131(40)	196(60)	1.05(0.39–2.82)	0.927
Use of protective eye wear				
Yes	122(40)	184(60)	1.10(0.55–2.17)	0.791
No	16(42)	22(58)	1	
Duration of welding(years)				
≤ 5	60(47)	69(53)	1	
6–10	38(42)	53(58)	1.21(0.71–2.08)	0.485
> 10	39(32)	84(68)	1.87(1.12–3.13)	0.131
History of ocular trauma				
Yes	93(41)	135(59)	0.92(0.58–1.45)	0.721
No	45(39)	71(61)	1	
Type of protective eye wear				
sun glasses	128(40)	189(60)	0.33(0.07–1.54)	0.158
Others	2(18)	9(82)	1	
History of Foreign body removal				
Yes	67(36)	119(64)	1.45(0.94–2.24)	0.093
No	71(45)	87(55)	1	

*statistically significant at 5% confidence level

At multivariate analysis (Table 5), the female welders were 4.3 times more likely to have an ocular disorder, as compared to the male counterparts, keeping other factors constant (AOR = 5.414, *P*-value = 0.005). Welders, 35 years and above were 4.2 times more likely to have an ocular disorder keeping other factors constant (AOR = 4.244, *P* value = < 0.001)Those who reported a history of foreign body removal were 1.7 times more likely to have an ocular disorder, as compared to those who had no such history, keeping other factors constant (OR = 1.677, *P*-value = 0.041).

**Table 5:** Multi variable logistic model for the factors associated with ocular disorders among small scale welders

Variable	Adjust OR	(95%CI)	*p*-value
Sex			
Male	1		
Female	4.279	(1.494–12.253)	0.007*
Education level			
none/primary	1		
secondary/tertiary	0.867	(0.534–1.407)	0.564
Use of protective eye wear			
No	1		
Yes	1.118	(0.516–2.421)	0.778
Foreign body removal			
No	1		
Yes	1.677	(1.023–2.751)	0.041*
Age			
less than 35 years	1		
35 years and above	4.244	(2.328–7.737)	< 0.001*
History of trauma			
No	1		
Yes	1.205	(0.711–2.041)	0.469
Duration of welding			
≤ 5 years	1		
6–10 years	1.140	(0.625–2.081)	0.669
> 10 years	0.849	(0.420–1.717)	0.649

*statistically significant at 5% confidence level

## Discussion

We found that the majority of the participants were male, and about 3 in every 5 of them had ocular disorders. Females, age 35 and above, and history of foreign body removal were significantly associated with ocular disorders.

The overall prevalence of ocular disorders among small-scale welders in Katwe, Kampala was found to be 59.88%, comparable, though slightly lower than the 66.4% prevalence found in a study done in Nigeria [19]. This could be due to a difference in climate in the different countries, at different times of the year, as the commonest disorders in both groups were conjunctival disorders, which are more prevalent in hot windy climates. Several studies have been conducted in the general population that show prevalence of specific ocular disorders but none gives information about overall prevalence especially in our Ugandan setting.

Conjunctiva disorders 128 (32.0%) and Presbyopia 94(27.0%), were the commonest ocular findings in this study, followed by the refractive error and corneal disorders. The commonest disorder was pingueculum (13%). This is similar to other studies, in that pingueculum was the commonest disorder, although the prevalence is lower than in similar studies [7, 19].

Pterygium was found in 27 participants (8%), which is higher than the general population [23], but lower than other studies. Welders have been noted to have a greater predisposition to developing pingueculum than pterygium [7]. Pterygium can cause visual impairment [24]. Conjunctival injection was the second commonest disorder, and was mostly due to irritants in the environment (dust), and other allergic reactions. No other studies reported this finding. This could be because it may have been considered under other conditions, such as conjunctivitis and keratitis.

Papillae, due to allergic conjunctivitis, were found in 16 (5%) participants. This is higher than in a similar study done in Nigeria, where the prevalence was 1.2% [7]. This could be due to a higher chance of exposure to allergens at the roadside workshops, in comparison to the above study, where even industrial welders were included. This is comparable to the prevalence 6–30% in the general population [25].

Presbyopia was found in 27% of the participants, 81.9% of whom were over 40 years of age. This prevalence is comparable, although lower than a similar study in welders, which found presbyopia in 36.8% of the participants [7]. The higher prevalence could be because the study had an older population, with a mean age of 39 years, as compared to this study, where participants had a mean age of 36 years. A study done in Tanzania showed a prevalence of 61.7% in the general population of participants who were 40 years and above, which is higher than is in this study. This is probably because the study had older participants [26]. The expected onset of presbyopia is 40 years, and incidence increases with age [26], which further explains the slightly lower prevalence in this study.

Refractive errors were found in 22 (6%) participants, 21 being myopic and 1 hyperopic. This is lower than other studies done in welders, where 25% of welders were found to have refractive errors [7]. This could be due to a difference in definition of refractive error; in this study, a refractive error was diagnosed only in patients with visual acuity less than 6/6, while other studies based on autorefraction findings only. This is comparable to global data that shows a prevalence of refractive errors of 7.2% [27]. In another study done in Southwestern Uganda [28], refractive errors were found in 13% of the participants who had poor vision; this finding in the general population, is comparable to the findings of this study, where refractive errors accounted for 10.6% of the ocular disorders.

Corneal abnormalities were found in 18 participants and these included corneal scars, keratitis, corneal tear, corneal erosion & ulceration, and trantas spots. All these conditions are potentially blinding. Studies have shown that non-ionizing radiation from welding can cause a variety of corneal disorders, such as keratitis [3]. Furthermore, welders are at higher risk of traumatic injury, accounting for the corneal scars, ulceration, erosion and tear [12].

From this study, female welders are more likely to have an ocular disorder, as compared to their male counterparts. This could be because welding is a male-dominated field, and females trying to engage in it may not have sufficient training and skill, to do the work. Other studies done did not have a significant number of female welders, thus this relationship was not explored [7].

Welders aged 35 years and above were 4.2 times more likely to have an ocular disorder than those younger. This could be because pingueculum, pterygium and presbyopia, some of the commonest ocular disorders found in the study, increase in prevalence with age. Presbyopia mainly occurs after the age of 40 and incidence increases with age [26]. Studies have also shown that pterygium prevalence increases with age [29–31]. Furthermore, welders who were aged more than 35 years were more likely to have worked for more than 10 years (results not shown) and thus longer duration of exposure to ultraviolet radiation emitted from welding. Long-term exposure to ultraviolet radiation is associated with conditions like such as pterygia, pingueculae, keratopathy, maculopathy and eye irritation [7, 8] which were ocular disorders found in this study.

Welders, who reported a history of foreign body removal, were 1.7 times more likely to have an ocular disorder, as compared to those who had none. This could be because ocular foreign bodies can cause a variety of disorders, such as; conjunctival hyperemia, corneal ulcers and scarring. Furthermore, welders who had exposure to ocular foreign bodies could have concurrently been exposed to other damaging factors from welding, for example, radiation that can cause ocular disorders. Though other studies reported a high prevalence of a history of ocular foreign bodies in welders, none explored the relationship between foreign bodies and ocular disorders [12, 32, 33].

The limitation to our study is that only small-scale welders were recruited for this study. For the study to be generalized to the entire welding community, industrial welders needed to be included.

## Conclusion

The prevalence of ocular disorders among the study participants was high. The most common disorders found were conjunctival disorders, presbyopia and myopia. Being female, age of 35 and above, and history of foreign body removal, were significantly associated with ocular disorders.

Findings from this study are generalizable to other small scale welders in many other low income countries, which have similar working conditions.

Welders should be given regular eye checks, and educated about the different potential ocular hazards related to their occupation.

There is a need for policies, to ensure that all welders use proper personal protective equipment (welding helmets).

A wider study that includes industrial welders is needed, to get results that can be generalized to the entire welding community.

## Additional file

Additional file 1: Questionnaire to Assess the prevalence, pattern and factors Associated with ocular disorders among small scale Welders in Katwe, Kampala. (DOCX 42 kb)

## Data Availability

All data and materials are available upon reasonable request from the author. Other requests for data can be made through the chair School of Medicine Research Ethics Committee, available at rresearch9@gmail.com.

## Abbreviations

PPE: Personal Protective Equipment
