# Supplementary material for: Prevalence, pattern and factors associated with ocular disorders in small-scale welders in Katwe, Kampala
Source: BMC Ophthalmol. 2019 Jul 10;19:145. doi: 10.1186/s12886-019-1150-x (PMC6617680; doi:10.1186/s12886-019-1150-x)
Supplement: Supplementary file 1 — Questionnaire to Assess the prevalence, pattern and factors Associated with ocular disorders among small scale Welders in Katwe, Kampala. (DOCX 42 kb) [file 12886_2019_1150_MOESM1_ESM.docx]

**DATA COLLECTING TOOL**

**TITLE: QUESTIONNAIRE TO ASSESS THE PREVALENCE, PATTERN AND FACTORS ASSOCIATED WITH OCULAR DISORDERS AMONG SMALL SCALE WELDERS IN KATWE, KAMPALA.**

**Quality Control**

Participant’s ID No: Date of Evaluation: _____/_____/________

Day/Month/Year

Interviewer Initials: Start Time: End Time

Phone……………………..

1. **DEMOGRAPHIC DATA**

**1.** Age(in years)…………………………

**2.** Sex [ ] 1. Male 2. Female

3. Educational level [ ] 1. None 2. Primary 3. Secondary 4. Tertiary

1. **CLINICAL HISTORY**
2. Type of welding [ ] 1. Gas flame 2. Electric Arc
3. Use of protective eye wear [ ] 1. yes 2. No
4. Duration of welding (in years)………………………………
5. History of ocular trauma [ ] 1. yes 2. No
6. If yes; Agent causing injury[ ] 1. flying metal chips 2. Flames 3. Radiation

4. Others, (specify)………………………………………………..

1. Did injury happen while using protective eye wear? [ ] 1. yes 2. No
2. Type of protective eye wear [ ] 1. welding helmet with filters

2. sun glasses 3.Others, (specify)………………………………………………….

1. Do you use spectacle correction for seeing? [ ] 1. Yes 2. No
2. Do you use your spectacle correction (if any) during welding [ ] 1. yes 2. No
3. History of foreign body removal [ ] 1.yes 2. No

**C .EXAMINATION:**

|  | Right | Left |
| --- | --- | --- |
| Visual acuity: | [ ] 1=6/4 2=6/5 3=6/6 4=6/12 5=6/18 6=6/36 7=6/60 8=CF5M 9=CF4M 10=CF3M 11=CF2M 12=CF1M 13=CFNear 14= HM 15=PL 16=NPL | [ ] 1=6/4 2=6/5 3=6/6 4=6/12 5=6/18 6=6/36 7=6/60 8=CF5M 9=CF4M 10=CF3M 11=CF2M 12=CF1M 13=CFNear 14= HM 15=PL 16=NPL |
| Stereopsis | [ ] 1. yes 2. No | [ ] 1. yes 2. No |
| Visual field | [ ] 1.Normal 2.Abnormal | [ ] 1.Normal 2.Abnormal |
| Extaocular muscle activity | [ ] 1. yes 2. No | [ ] 1. yes 2. No |
| Diplopia | [ ] 1. yes 2. No | [ ] 1. yes 2. No |
| Intraocular pressure | [ ] 1. Low(˂10mmHg)  2. Normal (10-21mmHg)  3. High (>21) | [ ] 1. Low(˂10mmHg)  2. Normal (10-21mmHg)  3. High (>21) |
| Refractive error | [ ] 1.Normal 2.Myopia 3.Hypermetropia | [ ] 1.Normal 2.Myopia 3.Hypermetropia |
| presbyopia | [ ] 1. yes 2. No | [ ] 1. yes 2. No |
| **WHOLE GLOBE** | [ ] 1. Normal 2. Phthisis 3. Removed  Others, specify…………………………….. | [ ] 1. Normal 2. Phthisis 3. Removed  Others, specify…………………………….. |
| **EYE LIDS** | [ ] 1. Normal 2. Ptosis 3. Lid tear 4. Ectropion 5. Entropion 6. Others, specify ……………………………………. | [ ] 1. Normal 2. Ptosis 3. Lid tear 4. Ectropion 5. Entropion 6. Others, specify ……………………………………. |
| **CONJUNCTIVA** | [ ] 1. Normal 2. Injection 3. pingueculum 4. Pterygium 5. Foreign body 6. Others, specify………………………………. | [ ] 1. Normal 2. Injection 3. pingueculum 4. Pterygium 5. Foreign body 6. Others, specify………………………………. |
| **CORNEA** | [ ] 1. Normal 2. Erosion 3. Foreign body 4. Perforation 5. Perforation/tear 6. Opacity 7. Ulcer 8. Others, specify………………………………….. | [ ] 1. Normal 2. Erosion 3. Foreign body 4. Perforation 5. Perforation/tear 6. Opacity 7. Ulcer 8. Others, specify………………………………….. |

|  | Right | Left |
| --- | --- | --- |
| **ANTERIOR CHAMBER** | [ ] 1.Normal 2.Hyphema 3.Hypopyon 4.Foreign body 5.Others,specify……………………… | [ ] 1.Normal 2.Hyphema 3.Hypopyon 4.Foreign body 5.Others,specify……………………… |
| **IRIS** | [ ] 1. Normal 2. Rapture 3. Ciliary body damage 4. Choroidal damage 5. Others, specify……………………………… | [ ] 1. Normal 2. Rapture 3. Ciliary body damage 4. Choroidal damage 5. Others, specify……………………………… |
| **LENS** | [ ] 1. Clear 2. Partial opacity 3. Total opacity 4. Aphakia 5. Pseudophakia 6. Lens damage 7. Others, specify…………………………………. | [ ] 1. Clear 2. Partial opacity 3. Total opacity 4. Aphakia 5. Pseudophakia 6. Lens damage 7. Others, specify…………………………………. |
| **VITREOUS** | [ ] 1. Normal 2. Vitreous opacity 3. Vitreous hemorrhage 4. Others, specify………………………………… | [ ] 1. Normal 2. Vitreous opacity 3. Vitreous hemorrhage 4. Others, specify………………………………… |
| **OPTIC DISC** | [ ] 1. Normal 2. Cupped 3.Atrophy 4. Others, specify…………………………….. | [ ] 1. Normal 2. Cupped 3.Atrophy 4. Others, specify…………………………….. |
| **RETINA** | [ ] 1. Normal 2. retinitis 3. Retinal detachment 4.others,specify……………………………… | [ ] 1. Normal 2. retinitis 3. Retinal detachment 4.others,specify……………………………… |
| **MACULAR** | [ ] 1. Normal 2. Maculopathy 3. Others, specify………………………… | [ ] 1. Normal 2. Maculopathy 3. Others, specify………………………… |
